# Integrating Camera-Trap Monitoring and Community Surveys to Assess Human–Asian Badger (*Meles leucurus*) Interactions in Agricultural Landscapes of the Meng Mountains, China

**DOI:** 10.3390/ani16203294

**Published:** 2026-10-19

**Authors:** Kamal Ahmed Khan, Tengteng Liu, Zartasha Gul, Shaofei Shi, Changshan Shao, Jianhua Gong, Zenglei Shi, Guiwen Yang

**Affiliations:** 1 College of Life Sciences, Shandong Normal University, Jinan 250014, China; 2019200017@stu.sdnu.edu.cn; 2 Department of Zoology, Government Girls’ Degree College, Khalabat Township, Haripur 22620, Pakistan; 3 Mengyin County State-Owned Forest Farm, Linyi 276000, China; 4 Yimengshan World Geopark Administration, Mengshan Conservation and Development Center, Linyi 276000, China

**Keywords:** mesocarnivore, human–wildlife conflict, reported crop depredation, diel activity, rural livelihoods, public perception, coexistence

## Abstract

Human–wildlife interactions can threaten rural livelihoods and complicate wildlife conservation in agricultural landscapes. We combined community surveys and camera-trap monitoring as complementary sources of information to assess human–Asian badger (*Meles leucurus*) interactions across four sites in the Meng Mountains, China. We surveyed 400 residents and monitored 82 camera-trap stations from April 2024 to August 2026, representing 72,406 trap-nights. Reported encounter frequency differed significantly among study sites (χ^2^ = 139.36, *p* < 0.001), with evening and autumn most frequently reported. Agricultural land ownership was associated with reported crop damage (χ^2^ = 16.64, *p* = 0.0002), while livestock ownership was associated with both reported crop damage (χ^2^ = 36.23, *p* < 0.001) and conflict awareness (χ^2^ = 60.47, *p* < 0.001). Camera traps recorded 103 independent detections at 52 of 82 targeted stations (detection proportion = 0.63). Because stations were selected where badger occurrence was suspected, this proportion should not be interpreted as landscape-wide occupancy. Detections differed among diurnal, crepuscular, and nocturnal periods (χ^2^ = 14.99, *p* < 0.001), with greater representation during diurnal and crepuscular periods. Together, the ecological and questionnaire data provide complementary evidence on badger occurrence, agricultural exposure, livelihood activities, and human perceptions relevant to reported human–badger conflict. Integrating ecological monitoring with community information may help inform targeted, non-lethal conflict mitigation and coexistence planning.

## Simple Summary

Human–wildlife interactions can challenge both wildlife conservation and rural livelihoods, especially where farms are close to wildlife habitats. We investigated interactions between local communities and the Asian badger (*Meles leucurus*) in the Meng Mountains, China, using questionnaires from 400 residents and motion-triggered cameras at 82 locations. Reported encounters varied by site and most often occurred in the evening and autumn. Agricultural land ownership was associated with reported crop damage, while livestock ownership was associated with both reported crop damage and conflict awareness. Cameras recorded Asian badgers at 52 of 82 targeted locations and produced 103 separate detections. Badgers were detected more often during daytime and around dawn and dusk than at night. Together, the ecological and questionnaire data provide complementary information on badger occurrence, reported damage, and conflict awareness, without implying that encounters or badger occurrence alone constitute human–wildlife conflict. Combining wildlife monitoring with local community information may help inform targeted, non-lethal mitigation and coexistence planning.

## 1. Introduction

Human–wildlife conflict (HWC) occurs when interactions between people and wildlife produce negative consequences for human interests, wildlife, or both [1]. In agricultural landscapes, such interactions may include crop damage, livestock depredation, property damage, and other livelihood losses that can affect rural livelihoods and attitudes toward wildlife [1,2,3]. Because the occurrence and consequences of conflict vary among species and local contexts, effective management requires attention to species-specific ecological and socioeconomic conditions [2,3]. Human–badger interactions may become conflictual where badgers use agricultural and peri-rural habitats and their foraging or burrowing activities overlap with human land use. Asian badgers are opportunistic omnivores whose diets include plant material, fruits and seeds, invertebrates, and small vertebrates. In China, Asian badgers have been reported to forage on maize, potato, and sweet potato crops, indicating a direct potential for crop damage where foraging overlaps with cultivated land [4]. Studies of the European badger (*Meles meles*), a closely related congener, further show that sett construction and burrowing can alter soil conditions, increase herbaceous plant richness, facilitate seed dispersal, and contribute to local habitat heterogeneity [5]. The Asian badger (*Meles leucurus*) occupies heterogeneous landscapes that include natural habitats, agricultural areas, and other human-used environments [6]. However, the presence of agricultural land does not necessarily imply greater badger use or more frequent human–badger encounters. Asian badgers have been reported to select sett sites associated with deciduous forest, dense vegetation, and nearby structural cover, with lower sett abundance in non-forest and human-disturbed areas [7]; therefore, encounter frequency may depend on habitat configuration and disturbance intensity rather than agricultural cover alone. Understanding where and when Asian badgers occur, and under what circumstances their activities are associated with reported damage or conflict, is therefore important for developing management approaches that balance species conservation with rural livelihood concerns [6]. Recent camera-trap work in central North China indicates that Asian badger activity can vary with human disturbance, including reduced daytime activity where human disturbance is greater [6]. However, ecological information on *M. leucurus* in China remains geographically limited, particularly in agricultural landscapes where badger occurrence and human livelihood activities overlap [3,6]. For Asian badgers, conflict may arise where their use of human-modified landscapes overlaps with agricultural activities and other livelihood practices. Although recent studies have improved understanding of Asian badger occurrence, abundance, habitat characteristics, and responses to human disturbance in China, the links between ecological patterns and household-level exposure to wildlife remain less well understood [8,9]. Research on other conflict-prone wildlife in China has shown that integrating information on species distribution, activity, and damage can provide a stronger basis for targeted conflict mitigation [10]. Conflict outcomes are also influenced by how local people perceive wildlife, because tolerance toward species and support for management can vary with perceived costs, benefits, and previous experience [11]. In Chinese human–wildlife conflict systems, greater economic losses have also been associated with less favorable attitudes toward conflict-associated species, emphasizing the need to consider livelihood impacts alongside ecological information [12]. An assessment focused only on wildlife occurrence may therefore overlook the socioeconomic conditions that determine whether wildlife presence develops into perceived or experienced conflict. Conversely, questionnaire-based information on encounters and losses can be strengthened when interpreted alongside independently collected ecological monitoring data [10,11].

Developing effective coexistence strategies therefore requires consideration of both wildlife ecology and the social and livelihood conditions that shape people’s interactions with wildlife [11]. Ecological monitoring and community-based information can provide complementary perspectives on such interactions: field-based monitoring documents wildlife occurrence and activity, whereas social surveys capture human encounters, perceived impacts, and attitudes toward wildlife [6,10,13,14]. Camera traps are particularly useful for documenting the occurrence and temporal activity of elusive mammals without direct animal handling, providing ecological information that can be compared with patterns reported by local communities [9,10]. Combining these approaches can therefore help distinguish between where and when wildlife is detected and where people perceive or experience interactions, providing a broader basis for interpreting human–wildlife conflict [10,13,14]. The Meng Mountains of eastern China provide the geographical setting for this assessment [15].

This study combines camera-trap monitoring and community surveys as complementary approaches to examine the ecological and human dimensions of Asian badger (*Meles leucurus*) interactions across four sites in the Meng Mountains, China. Specifically, we combine ecological information on Asian badger occurrence and temporal activity with household-level information on reported encounters, agricultural impacts, socioeconomic characteristics, and perceptions of the species. The following are the study’s objectives: (1) to characterize Asian badger occurrence and temporal activity across the study area using camera-trap records, together with spatial and temporal patterns in community-reported encounters; (2) to examine associations between reported human–badger interactions and socioeconomic characteristics, including agricultural land use and livestock ownership; (3) to assess reported Asian badger-related crop damage and associated economic losses and to evaluate factors associated with variation in reported damage; and (4) to examine respondent familiarity, perceptions, and conservation-related awareness of Asian badgers and their associations with reported human–wildlife interactions. Together, these components provide an integrated assessment of Asian badger ecology and human interactions in a mountainous agricultural landscape, with implications for locally targeted conflict mitigation and conservation management.

## 2. Materials and Methods

### 2.1. Study Area

**Figure 1:**
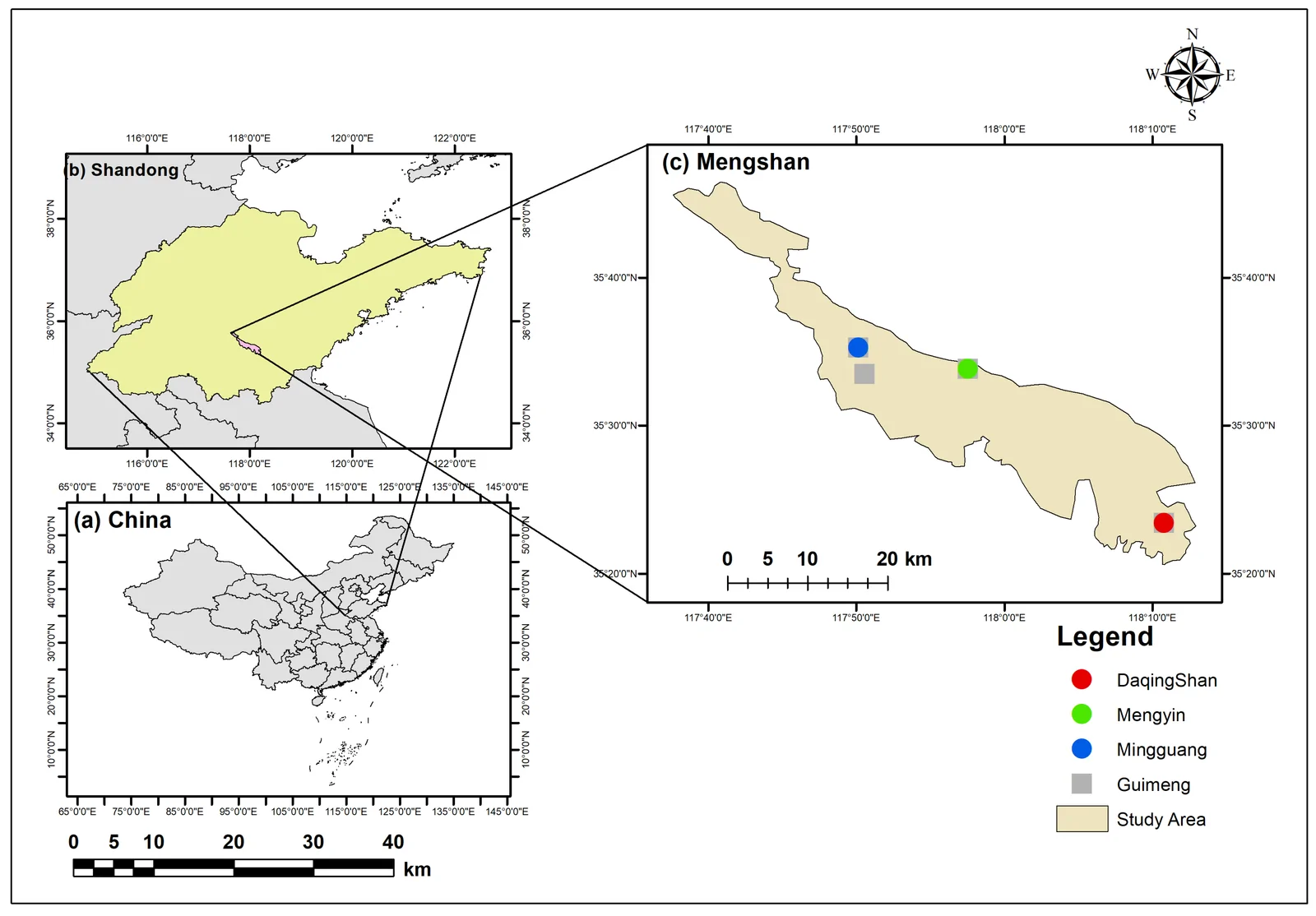
Location of the four study sites in the Meng Mountains, Shandong Province, China. (**a**) Location of Shandong Province within China; (**b**) location of the Meng Mountains within Shandong Province; and (**c**) locations of Mingguang, Mengyin, Guimeng, and Daqingshan study sites.

The study was conducted at four sites in the Meng Mountains, Shandong Province, eastern China: Mingguang Forest Farm in Pingyi County, Tianma Forest Farm in Mengyin County, Guimeng/Wanshougong Forest Farm in Pingyi County, and Daqingshan Forest Farm in Fei County (Figure 1). We selected these sites to represent variation in human population density, agricultural practices, and proximity to natural habitats used by the Asian badger (*Meles leucurus*). The Meng Mountains cover approximately 240 km^2^ and comprise a heterogeneous landscape of forest, shrubland, agricultural land, and human-use areas. Pine and poplar stands dominate the vegetation, while shrubs and herbaceous vegetation are widespread on slopes and in valleys and cover about 70% of the area. Elevation across the study area ranges from approximately 400 to 1000 m above sea level [15]. We conducted questionnaire surveys among residents associated with the four study areas, while deploying camera traps primarily within the corresponding forest farms, with a smaller number positioned in nearby agricultural areas.

### 2.2. Camera-Trap Survey and Asian Badger Monitoring

**Figure 2:**
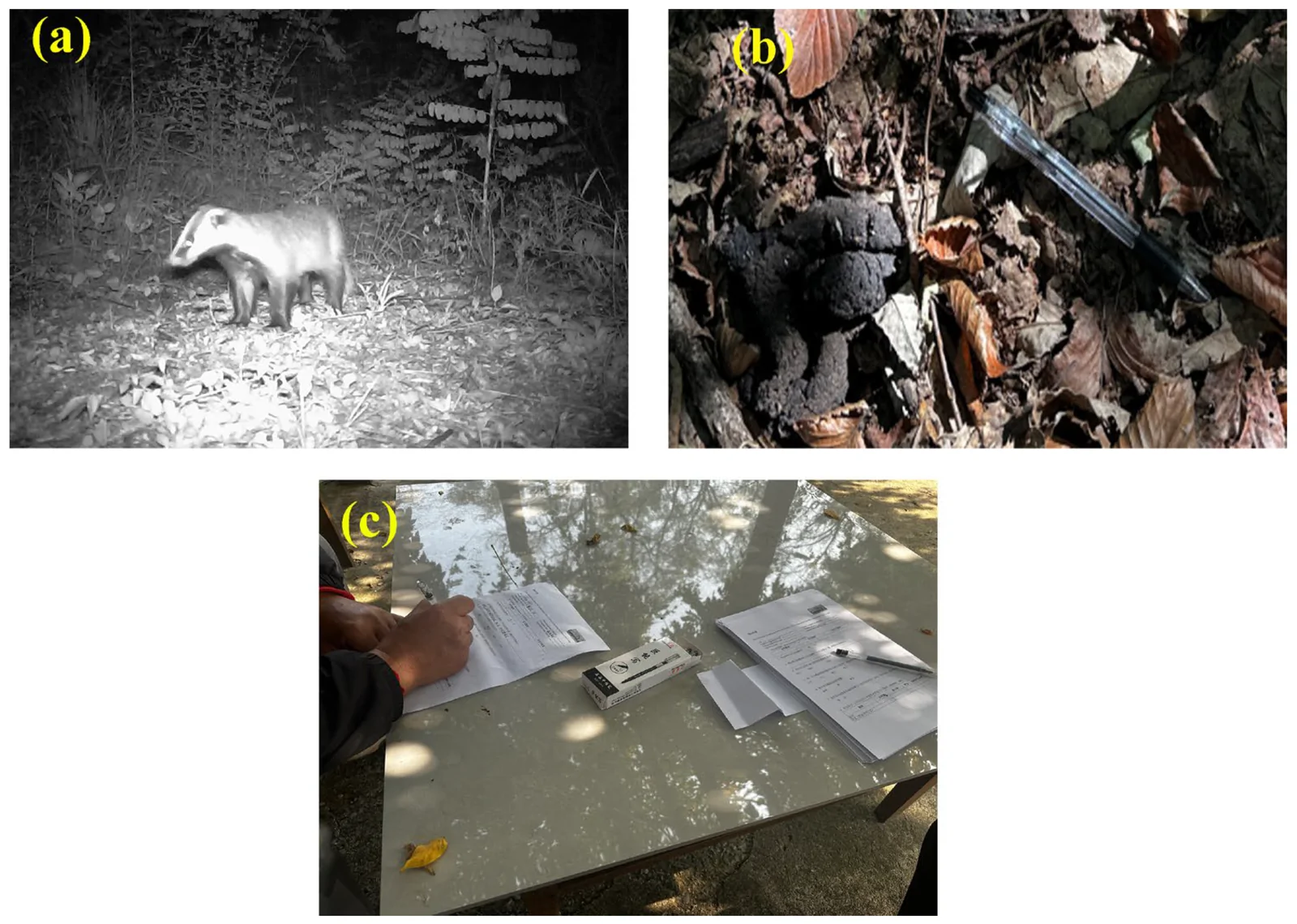
Field evidence and survey activities in the Meng Mountains. (**a**) Infrared camera-trap record of an Asian badger (*Meles leucurus*); (**b**) fecal deposits recorded at a camera-monitored location where Asian badger defecation was directly documented; no laboratory confirmation was performed; and (**c**) administration of the community questionnaire.

Preliminary field surveys identified suitable camera-trap locations and confirmed the presence of Asian badgers within the four study areas. Non-invasive methods included infrared camera traps (Yianws L710, version V1.0.032C; Shenzhen, China) and searches for indirect signs along potential badger movement routes. We recorded fecal deposits at monitored locations where camera-trap sequences directly documented Asian badgers defecating. No genetic, microscopic, or other laboratory confirmation of the fecal material was performed; therefore, fecal evidence was used only as supporting field evidence and not as an independent basis for confirming species presence (Figure 2).

After the preliminary survey, we established 82 camera-trap stations across the four study areas. We conducted monitoring from April 2024 to August 2026, comprising 275 sampling nights from April to December 2024, 365 nights from January to December 2025, and 243 nights from January to August 2026. The resulting sampling period was 883 nights per camera.

Operational trap-night effort was calculated from the documented camera deployment and monitoring records. All 82 camera traps remained deployed and operational throughout the monitoring period. Cameras were routinely inspected approximately 3–4 times per month; during periods of reduced winter activity, inspection intervals were extended to approximately one month while cameras remained active. During maintenance visits, camera function and battery levels were checked, and batteries were replaced before depletion. Camera-trap data were copied to a laptop, after which the SD cards were cleared and returned to the cameras. No camera failures, battery depletion, full memory cards, theft, damage, or other operational interruptions were recorded. Therefore, all 883 sampling nights were operational for each of the 82 cameras, yielding a total effective sampling effort of 72,406 trap-nights. We selected camera stations after preliminary surveys in areas where Asian badger occurrence was suspected. We recorded geographic coordinates and elevation for each camera-trap station using a Global Positioning System (GPS). We positioned cameras to minimize false triggers from foreground vegetation [16]. Camera height generally followed a conventional range of approximately 40–60 cm, though placement height was adjusted where needed to reduce camera visibility and theft risk.

To reduce temporal non-independence, we treated multiple Asian badger detections recorded at the same camera station within 30 min as part of the same event; detections separated by ≥30 min were considered independent events [17]. We defined a trap-night as a complete 24 h period during which a camera at a sampling station was operational. The Relative Abundance Index (RAI) was calculated as the number of independent Asian badger detections per 100 trap-nights [18,19]:

RAI = *a_i_*/*N* × 100

where *a_i_* is the total number of independent Asian badger detections, and *N* is the total number of operational trap-nights. We calculated the station-level detection proportion as the number of camera-trap stations at which Asian badgers were detected divided by the total number of monitored stations. Because camera stations were selected in areas where badger occurrence was suspected, this proportion is descriptive of the targeted camera network and should not be interpreted as an unbiased estimate of landscape-wide occupancy.

We analyzed diel activity using timestamps from independent Asian badger detections. We estimated the activity level, defined as the estimated proportion of time animals were active, by fitting kernel density functions to camera-trap detection times following Rowcliffe et al. [20]. We conducted analyses in R 4.5.2 using the activity package [21,22]. We applied the fitact function to the independent detection times to fit a circular kernel model and estimate the activity level; we used the sample = “data” argument to obtain a standard error. The 24 h cycle was divided into three activity periods: crepuscular, defined as one hour before to one hour after sunrise and sunset; diurnal, defined as one hour after sunrise to one hour before sunset; and nocturnal, defined as one hour after sunset to one hour before sunrise. Selection ratios were calculated to describe the relative use of each activity period [8,23,24].

*w_i_* = *o_i_*/*π_i_*

where *w_i_* is the selection ratio for activity period *i*, *o_i_* is the proportion of independent detections recorded during that period, and *π*_i_ is the proportion of the 24 h cycle represented by that period. Values of *w_i_* > 1 indicated greater use relative to availability, whereas values of *w_i_* < 1 indicated lower use.

### 2.3. Questionnaire Design and Data Collection

A structured questionnaire was developed to collect information on respondents’ experiences with Asian badgers, including familiarity with the species, sightings and encounter frequency, reported encounter timing and seasonality, conflict awareness, agricultural impacts, livestock ownership, economic losses, compensation, and perceptions of coexistence [13,14]. The questionnaire covered three broad domains: (i) respondent characteristics and land use; (ii) familiarity with Asian badgers, sightings, encounter patterns, and perceptions; and (iii) livestock ownership, reported crop damage, economic loss, compensation, and potential coexistence measures. Respondent familiarity was a self-reported subjective measure assessed by the question, “How familiar are you with the Asian badger (*Meles leucurus*)?”, with response options of “very familiar,” “somewhat familiar,” and “not familiar at all” (Supplementary Material S1: Questionnaire). No additional operational definition of familiarity was provided during questionnaire administration; therefore, this variable should be interpreted as respondents’ perceived familiarity rather than an objective measure of knowledge or experience. These questions characterized respondents’ reported experiences with Asian badgers and examined how encounter patterns, agricultural activities, livelihood characteristics, and perceptions varied among respondents and study sites. The complete questionnaire is provided as Supplementary Material S1. The questionnaire survey was administered from November 2024 to November 2025. Participants were recruited with assistance from local foresters and local contacts, who helped identify farmers, forestry personnel, and people engaged in wildlife-related work associated with the four study areas. Participation was voluntary. No refusals were recorded, and all recruited participants completed the questionnaire.

A total of 400 respondents participated in the questionnaire survey, with 100 respondents from each of the four study areas: Mingguang, Mengyin, Guimeng, and Daqingshan. Respondents reported whether they had observed Asian badgers in the wild, where sightings occurred, how many animals they observed during an encounter, and how often they encountered badgers. Question 2 asked whether respondents had ever seen an Asian badger “in the wild,” whereas Question 4 asked how frequently they encountered badgers “in your area” (Supplementary Material S1: Questionnaire). These questions were therefore treated as distinct self-reported measures rather than interchangeable indicators: the former recorded whether a respondent had ever observed the species in the wild, while the latter recorded reported local encounter frequency. We acknowledge that the different wording may nevertheless have been interpreted differently among respondents.

Respondents recorded the encounter frequency using five categories: daily, weekly, monthly, rarely, and never. Respondents also reported the time of day at which they most often observed badgers (morning, afternoon, evening, or night), with multiple responses permitted. They were further asked during which season they noticed greater badger activity, choosing among spring, summer, autumn, and winter. Conflict awareness was assessed by asking whether respondents were aware of human–badger conflicts in their area, including examples such as property damage or livestock predation. Throughout this study, we distinguish among badger occurrence, human–badger encounters, reported crop damage, and conflict awareness. An encounter refers to a respondent-reported sighting or observation of an Asian badger and is not, by itself, considered a conflict. Conflict awareness refers specifically to respondents’ responses to the questionnaire item asking whether they were aware of human–badger conflicts in their area, with examples including property damage or livestock predation. Importantly, conflict awareness and reported crop damage were treated as separate questionnaire variables. The conflict-awareness item asked, “Are you aware of any conflicts between badgers and humans in your area? (e.g., property damage, livestock predation)” with yes/no responses, whereas the crop-damage item was worded, “Crops damaged by Asian badger in the past 1 year:“ and recorded the season, crop type, and reported loss (Supplementary Material S1: Questionnaire). Reported crop damage was not incorporated into the operational definition of conflict awareness, and responses to the two items were not cross-validated. Consequently, respondents could report crop damage attributed to Asian badgers without necessarily responding “Yes” to the broader conflict-awareness item. The questionnaire also recorded observations and perceived status of selected wildlife species occurring in the study landscape.

Perceptions of conservation protection were assessed by asking whether respondents believed that the Asian badger population had increased as a result of protection within the park, with response options of yes, no, or do not know. Livestock ownership was recorded by livestock type (goats, sheep, cattle, and equine/other livestock), together with the number owned and reported vaccination status. Respondents were asked whether they had experienced crop damage during the preceding year that they attributed to Asian badgers (Supplementary Material S1: Questionnaire). The phrase “during the preceding year” referred to the 12 months immediately preceding each respondent’s questionnaire date. Because questionnaires were administered from November 2024 to November 2025, this represented a respondent-specific rolling 12-month recall period rather than a single fixed calendar year. Direct observation of a badger causing the damage was not required, and respondents could base their attribution on field signs or their own experience. No independent verification procedure was used to exclude other potential crop-damaging species. Therefore, these data are treated throughout as respondent-attributed, reported Asian badger-related crop damage rather than as independently confirmed damage caused by Asian badgers. For reported damage, we collected the season of damage, crop type, and estimated monetary loss in Chinese yuan (RMB). Respondents were also asked whether they had received compensation from governmental or non-governmental organizations and, where applicable, the amount of loss compensated. Additional questions addressed payments for livestock grazing within park boundaries, respondents’ views on possible human–badger coexistence, and any additional observations concerning badgers in their area. Together, these variables characterized reported human–badger encounters, perceived impacts, livelihood characteristics, and attitudes toward Asian badgers across the four study areas. We obtained informed consent from all respondents before participation.

### 2.4. Statistical Analysis

We processed and analyzed the questionnaire data using Python 3.12 [25], with Pandas (version 2.3.3) for data management and cleaning, NumPy (version 2.3.5) for numerical operations, and SciPy (version 1.16.3) for statistical analyses. We conducted camera-trap activity analyses in R, as described in Section 2.2. Descriptive statistics, including frequencies, percentages, means, and other summary measures where appropriate, were used to describe respondent characteristics, familiarity with Asian badgers, encounter frequency, reported encounter timing and seasonality, crop-damage reports, livestock ownership, compensation, economic loss, and perceptions of the species. Some questionnaire items were left unanswered by individual respondents. The reasons for item non-response were not systematically recorded. Analyses were therefore conducted using available cases for the variables included in each analysis, and the resulting sample size (N) is reported for each statistical test or model. Consequently, effective sample sizes differed among analyses depending on item-level response availability.

Questionnaire-derived variables used as coded predictors were assigned numerical values as follows: respondent familiarity was coded as 0 = not familiar at all, 1 = somewhat familiar, and 2 = very familiar; encounter frequency was coded as 0 = never, 1 = rarely, 2 = monthly, 3 = weekly, and 4 = daily; active time was coded as 0 = night, 1 = evening, 2 = afternoon, and 3 = morning; and active season was coded as 0 = winter, 1 = autumn, 2 = summer, and 3 = spring. We assessed potential outliers in continuous variables using the interquartile range (IQR) criterion of 1.5 × IQR. We evaluated normality using the Shapiro–Wilk test to guide the selection of parametric statistical procedures. We used independent-sample t-tests for comparisons between two groups. For comparisons involving more than two groups, we used a one-way analysis of variance (ANOVA), followed by Tukey’s honestly significant difference (HSD) test for post hoc comparisons where applicable. Descriptive statistics and one-way ANOVA were used to assess variation in respondent-reported encounter patterns by the time of day and season.

We used chi-square tests of independence to evaluate associations among categorical variables, including reported crop damage, agricultural land ownership, livestock ownership, compensation, conflict-related responses, and habitat- or sighting-related categories [26,27]. Pearson correlation coefficients were used to examine linear relationships between continuous variables where applicable [26]. In contrast, Spearman’s rank correlation assessed the association between respondent-reported encounter frequency and familiarity with Asian badgers [28]. Ordinal logistic regression examined predictors of ordinal response variables, including respondent familiarity and perception-related responses, using socioeconomic, protection-related, and encounter-related variables as predictors [25]. Multiple linear regression models examined relationships between reported encounter/distribution patterns and temporal variables and assessed predictors of reported crop damage, including familiarity and encounter-related variables [29].

Effect sizes were calculated where applicable, including Cohen’s d for two-group comparisons, eta-square η^2^ for ANOVA, and Cramér’s V for chi-square associations. Statistical significance was evaluated at α = 0.05, and multiple-comparison adjustments were applied to post hoc comparisons where appropriate.

## 3. Results

### 3.1. Respondent Characteristics

A total of 400 respondents completed the questionnaire, with 100 respondents from each of the four study areas: Mingguang, Mengyin, Guimeng, and Daqingshan (Table 1).

Respondents comprised 54% men and 46% women. Of the 400 respondents, 331 (82.75%) reported agricultural land ownership, while 48 (12.0%) reported owning at least one livestock animal. The reported mean agricultural land area across surveyed households was 2.5 ha.

### 3.2. Human-Reported Asian Badger Encounters, Familiarity, Conflict, and Compensation

#### 3.2.1. Self-Reported Respondent Familiarity

**Figure 3:**
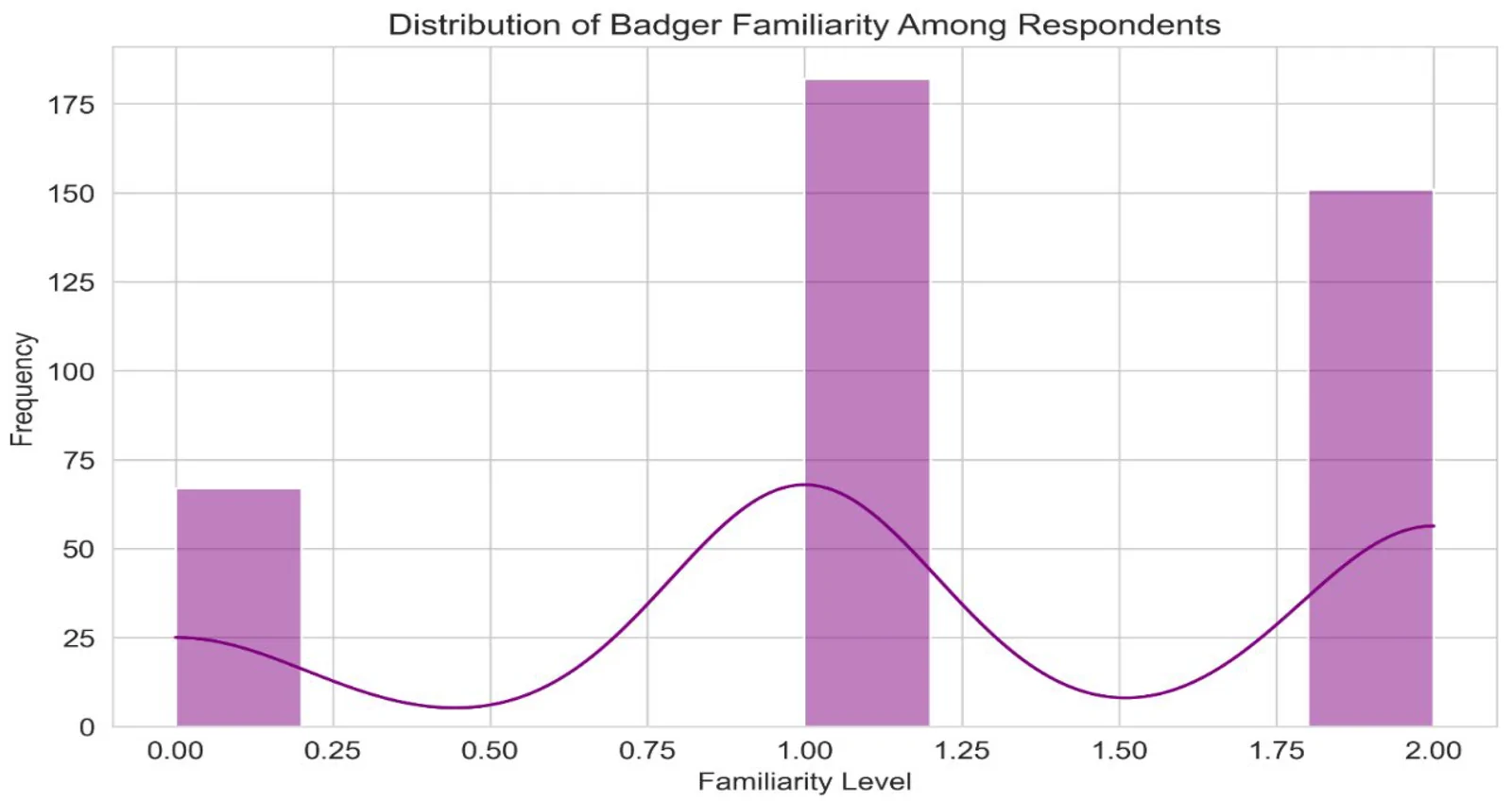
Respondent familiarity with Asian badgers (*Meles leucurus*) across the four study sites. Familiarity scores were coded as 0 = not familiar at all, 1 = somewhat familiar, and 2 = very familiar.

Respondents varied in their self-reported familiarity with Asian badgers. Of the 400 respondents, 180 reported being somewhat familiar, 150 reported being very familiar, and 70 reported being not familiar at all with the species (Figure 3).

#### 3.2.2. Spatial Variation in Reported Encounters

Using Question 4, respondents selecting daily, weekly, monthly, or rarely were classified as reporting encounters, whereas respondents selecting “never” were classified as not reporting encounters. Overall, 239 of 400 respondents (59.75%) reported encounters. The site-level proportions were 62% in Mingguang, 25% in Mengyin, 55% in Guimeng, and 97% in Daqingshan. Among respondents reporting encounters, weekly encounters were the most frequently reported category (55%). Reported encounter frequency differed significantly among study sites (χ^2^ = 139.36, *p* < 0.001; Cramér’s V = 0.417).

**Figure 4:**
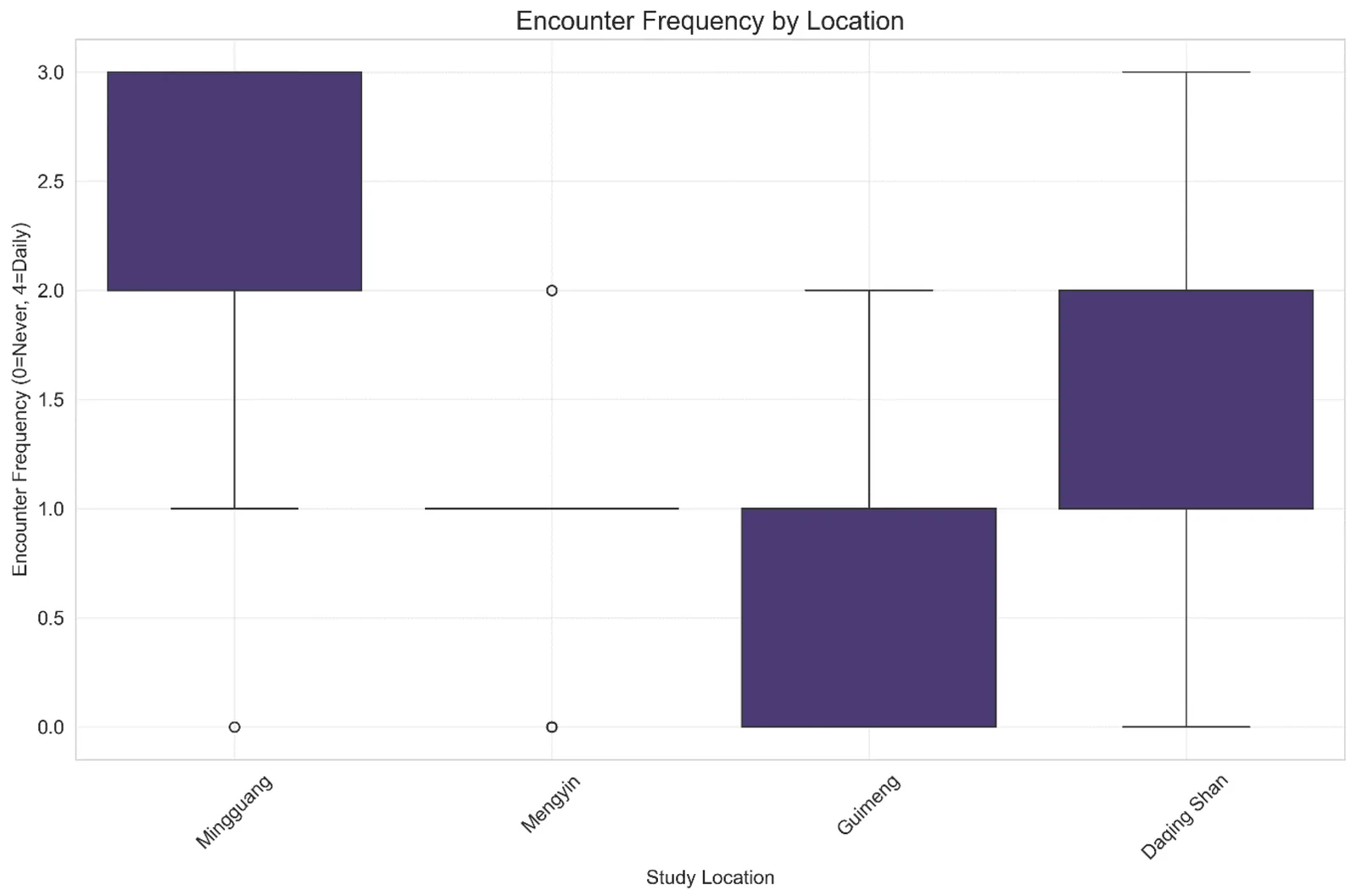
Boxplots showing the frequency of encounters between humans and Asian badgers in each of the four research regions (0 = never, 4 = daily). Separate points indicate outliers; the median and interquartile ranges are presented.

Reported encounter frequency also varied among the four study sites, with Mingguang showing the highest median encounter frequency and Mengyin the lowest, while Guimeng and Daqingshan showed intermediate values (Figure 4).

#### 3.2.3. Factors Associated with Self-Reported Familiarity

We used ordinal logistic regression to examine factors associated with respondents ’ self-reported familiarity with Asian badgers (Table 2). Perceived population increase associated with park protection (β=1.788, SE = 0.270, p<0.001) and encounter time (β=2.155, SE = 0.192, p<0.001) were significantly positively associated with self-reported familiarity, whereas active time was not statistically significant (β=0.275, SE = 0.146, p=0.059).

#### 3.2.4. Relationship Between Conflict Awareness and Encounter-Related Variables

We used Spearman’s rank correlations to examine the associations between respondents’ awareness of human–badger conflict and familiarity, encounter time, and active season (Table 3). None of these associations was statistically significant: familiarity (ρ = −0.002, *p* = 0.964), encounter time (ρ = −0.021, *p* = 0.676), and active season (ρ = −0.068, *p* = 0.178). Sample sizes varied according to data availability; analyses with complete responses used N = 400, whereas the analysis reported with N = 394 reflected six unavailable responses. We detected no significant monotonic associations between conflict awareness and these variables.

**Figure 5:**
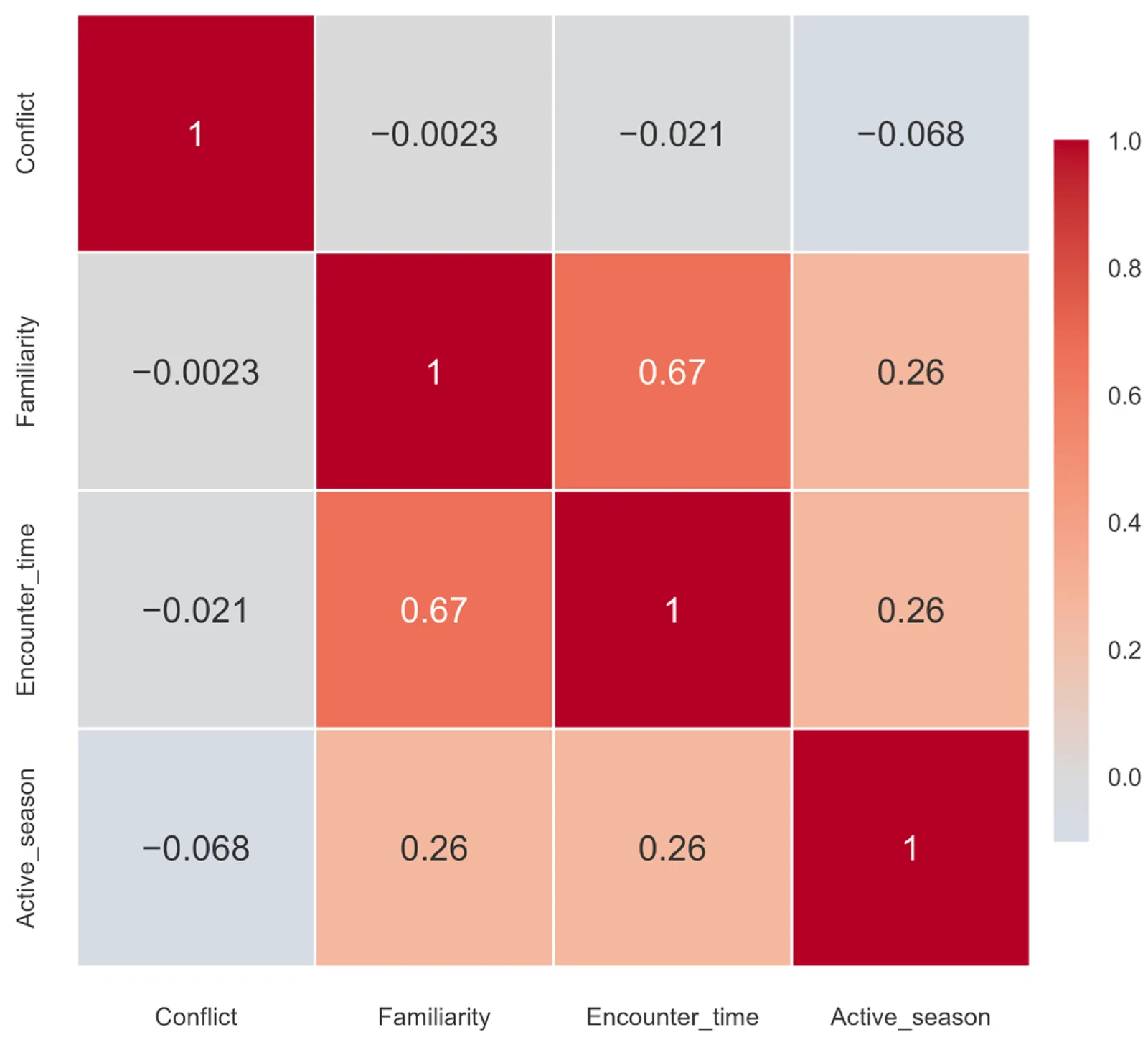
Spearman correlation matrix showing relationships among respondent familiarity, encounter time, active season, and conflict awareness.

The broader Spearman correlation matrix showed a positive association between encounter time and familiarity (ρ = 0.67), whereas the other pairwise associations were weaker (Figure 5).

#### 3.2.5. Crop Damage and Compensation

No significant association was detected between reported crop damage and receipt of compensation (χ^2^ = 0.000, *p* = 1.000). Observed and expected frequencies were identical, indicating no detectable difference in compensation status between respondents reporting crop damage and those reporting no crop damage. The corresponding Phi coefficient was 0.000.

### 3.3. Reported Crop Damage in Relation to Agricultural Characteristics and Spatial Variation

We used chi-square tests of independence to examine associations between agricultural characteristics and reported Asian badger-related crop damage. Agricultural land ownership was significantly associated with reported crop damage (χ^2^ = 16.64, df = 2, *p* = 0.0002; Cramér’s V = 0.204; Table 4). Respondents in the agricultural-land category reported crop damage more frequently than the respondents without agricultural land. Livestock ownership was also significantly associated with reported crop damage (χ^2^ = 36.23, df = 8, *p* < 0.001; Cramér’s V = 0.301; Table 4), with reported crop-damage frequencies differing among livestock-ownership categories.

Overall, 199 of the 400 respondents reported Asian badger-related crop damage, whereas 201 reported no crop damage. Among respondents reporting crop damage, 16 provided monetary estimates of crop losses; these data are presented separately in the economic-loss analysis.

**Figure 6:**
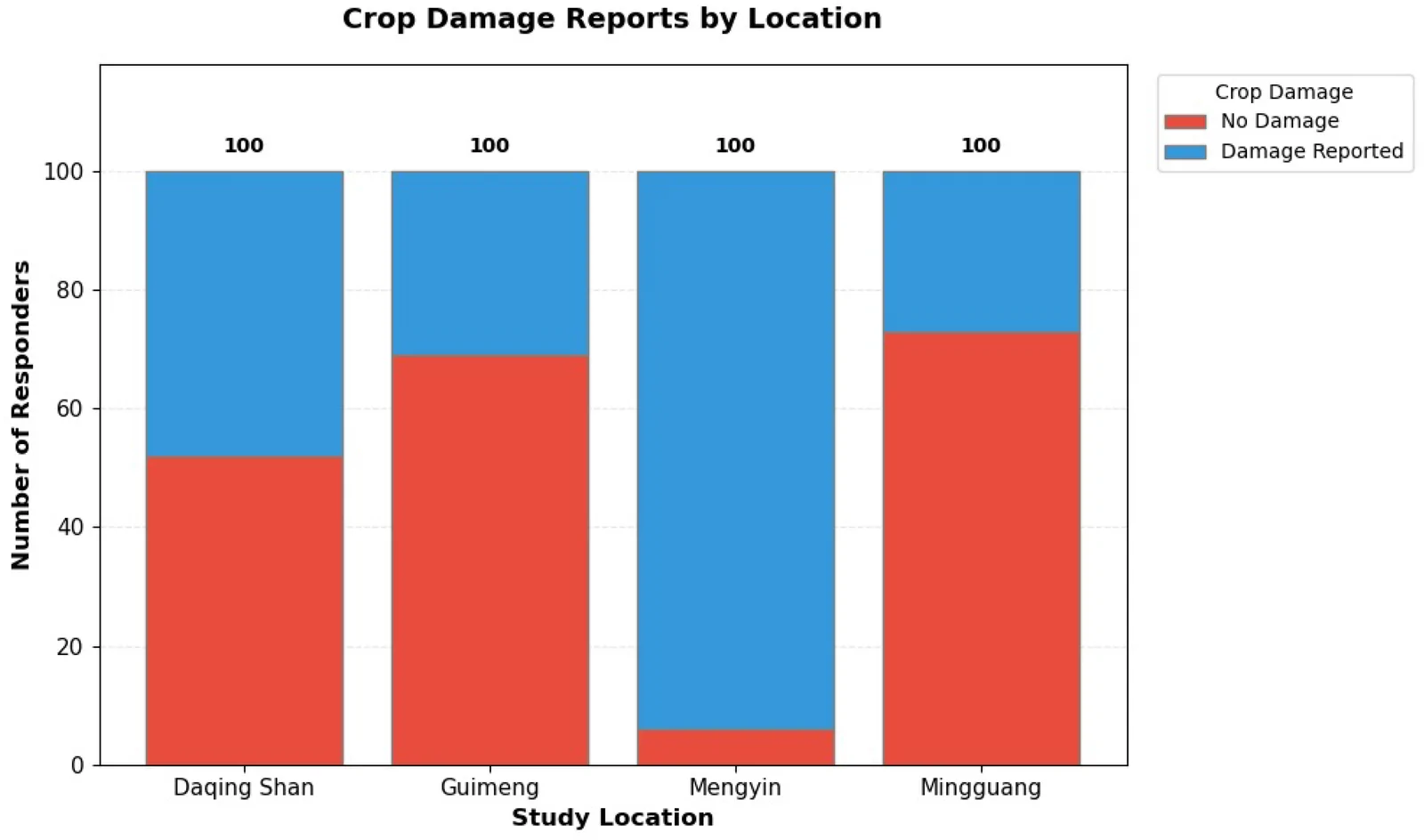
Number of respondents reporting Asian badger-related crop damage and no crop damage across the four study sites (n = 100 respondents per site). Red indicates no crop damage and blue indicates reported crop damage.

Reported crop damage occurred in all four study areas but varied substantially among sites (Figure 6). Mengyin had the highest proportion of respondents reporting crop damage (approximately 94%). Mingguang and Guimeng had lower proportions of reported crop damage, with approximately 74% and 69% of respondents, respectively, reporting no crop damage. Daqingshan showed an intermediate pattern, with approximately similar proportions of respondents reporting crop damage and no crop damage.

### 3.4. Spatial and Temporal Variation in Reported Asian Badger Encounters

**Figure 7:**
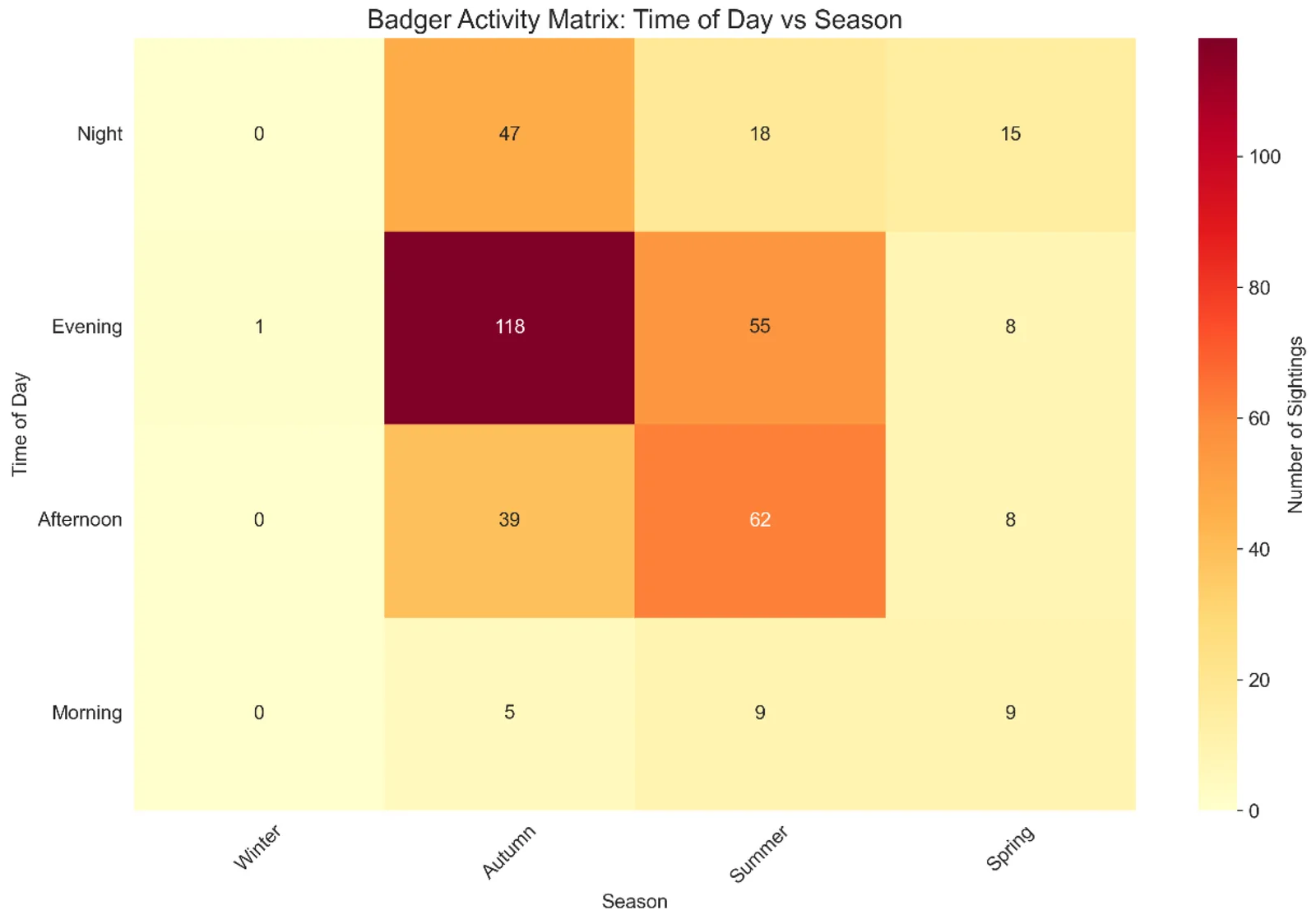
Heatmap of respondent-reported Asian badger (*Meles leucurus*) encounters by season and time of day. Darker cells indicate more reported encounters.

**Figure 8:**
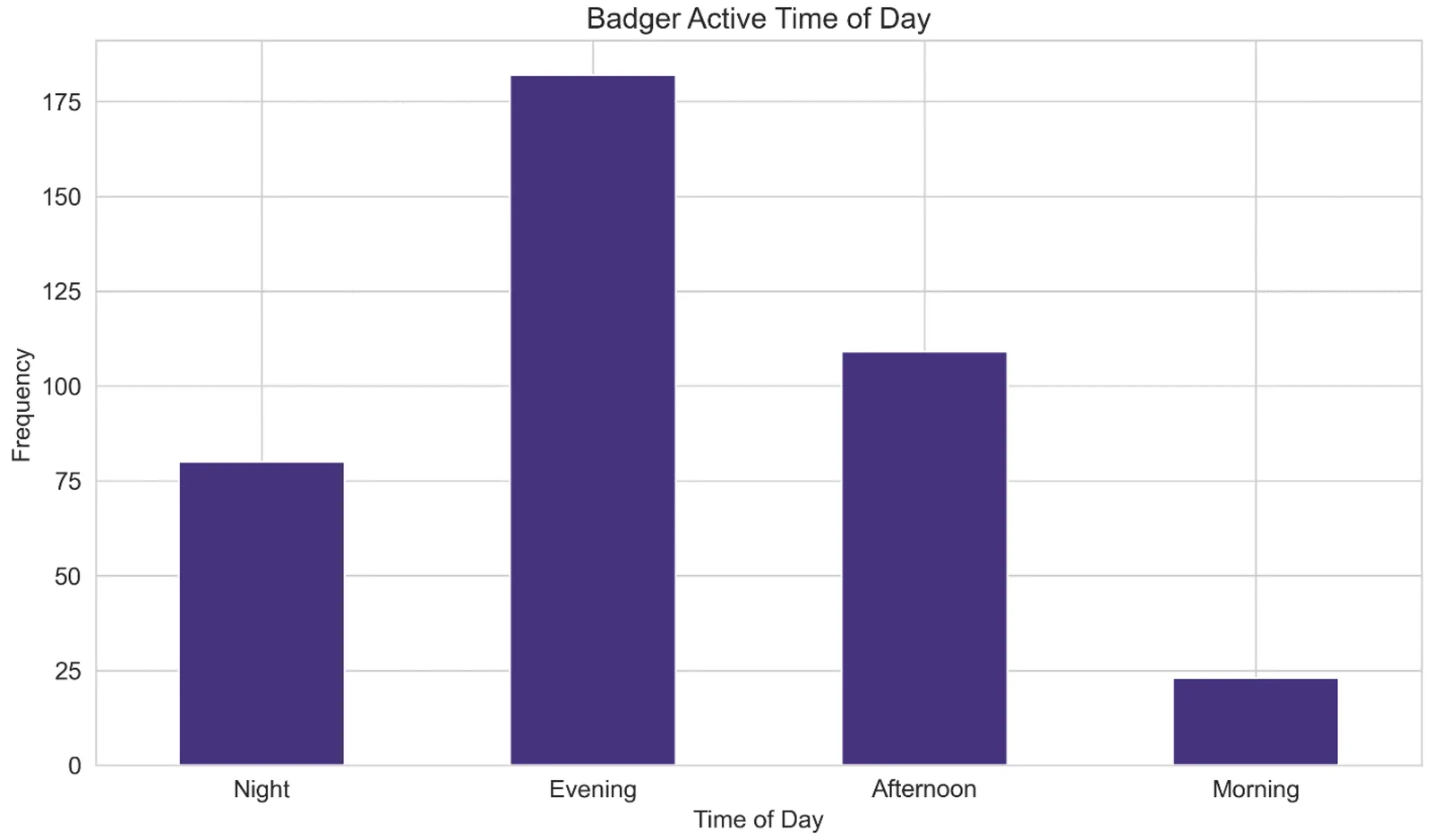
Frequency of respondent-reported Asian badger encounters across times of day. Evening encounters were most frequent, followed by afternoon and night encounters, whereas morning encounters were least frequent.

Respondent-reported Asian badger encounters varied across the time of day and season. Evening encounters were reported most frequently (N = 182), followed by afternoon encounters (N = 109), whereas morning and night encounters were less frequently reported (Figure 7 and Figure 8). Among the 394 respondents providing valid seasonal information, autumn was reported most frequently (N = 209), followed by summer (N = 144), spring (N = 40), and winter (N = 1; Table 5). Six respondents did not provide a response to the seasonal-activity item, resulting in 394 valid seasonal responses.

Spatial variation in reported encounters was also evident. A chi-square test indicated significant variation among sighting locations (χ^2^ = 139.357, df = 6, *p* < 0.001; Cramér’s V = 0.417; Table 6).

**Figure 9:**
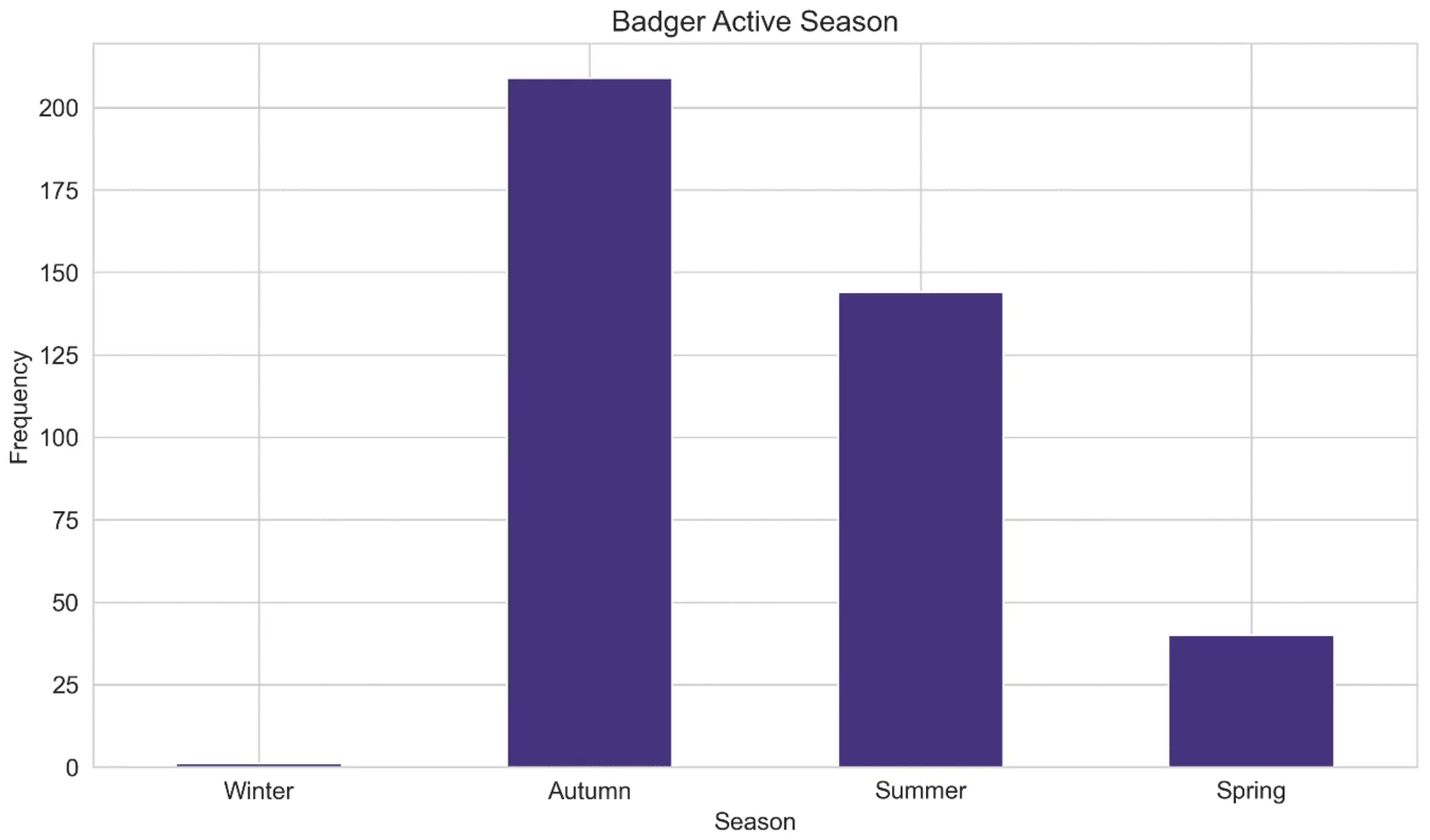
Seasonal distribution of respondent-reported Asian badger encounters. Reported encounters were highest in autumn, followed by summer and spring, and lowest in winter.

We used a multiple linear regression model to examine the associations between respondent-reported encounter frequency and encounter time, active time, and active season (Table 7). The model explained 28.9% of the variation in respondent-reported encounter frequency (R^2^ = 0.2888, adjusted R^2^ = 0.2834, *p* < 0.001; N = 394). Encounter time was positively associated with encounter frequency (β = 0.2729, *p* < 0.001), active season showed a negative association (β = −0.0575, *p* = 0.0425), and active time was not statistically significant (β = 0.0153, *p* = 0.491). Figure 9 summarizes seasonal variation in respondent-reported encounters.

### 3.5. Variation in Reported Asian Badger Encounters Across Spatial and Temporal Categories

The chi-square tests assessed variation in respondent-reported Asian badger encounters across location, time of sighting, and season of sighting (Table 8). The reported encounters differed significantly among locations (χ^2^ = 139.357, *p* < 0.001), times of sighting (χ^2^ = 16.007, *p* = 0.0137), and seasons of sighting (χ^2^ = 13.854, *p* = 0.0313).

### 3.6. Awareness, Self-Reported Familiarity, and Public Perceptions of Asian Badgers

We used an ordinal logistic regression model to examine factors associated with respondent ’ self-reported familiarity with Asian badgers (Table 9). The model was statistically significant overall (Pseudo R^2^ = 0.366, *p* < 0.001). Perceived population increase associated with park protection was positively associated with self-reported familiarity (β = 1.7884, SE = 0.2703, *p* < 0.001), as was encounter time (β = 2.1547, SE = 0.1924, *p* < 0.001). Active time of day was not statistically significant (β = 0.2752, SE = 0.1456, *p* = 0.0587).

A second ordinal logistic regression model examined associations between familiarity, encounter-related variables, and respondents’ perceptions of Asian badgers (Table 10). Familiarity was positively associated with public perceptions (β = 1.8376, SE = 0.2793, *p* < 0.001), whereas encounter time (β = 0.3289, SE = 0.2308, *p* = 0.1542) and active time of day (β = 0.2240, SE = 0.1836, *p* = 0.2224) were not statistically significant.

### 3.7. Livestock Ownership and Conflict Awareness

We evaluated the association between livestock ownership and respondents’ awareness of human–badger conflict using a chi-square test of independence. A significant association was detected between livestock-ownership categories and conflict awareness (χ^2^ = 60.474, df = 8, *p* < 0.001; Cramér’s V = 0.389; Table 11). Overall, 125 of 400 respondents reported awareness of human–badger conflict, whereas 275 did not report such awareness. Respondents without livestock made up the largest group in the survey (n = 352), and conflict awareness differed across livestock-ownership categories (Table 12).

### 3.8. Factors Associated with Reported Crop Damage

We used a multiple linear regression model to examine the associations between respondent familiarity, encounter time, active time, active season, and reported Asian badger-related crop damage (Table 13). The overall model was not statistically significant (R^2^ = 0.0215, adjusted R^2^ = 0.0009, *p* = 0.386; N = 195), and the included variables accounted for about 2.2% of the variation in reported crop-damage responses. Familiarity was not significantly associated with reported crop damage (β = −0.1382, *p* = 0.3031), nor was encounter time (β = −0.0804, *p* = 0.4756), active time (β = 0.0230, *p* = 0.7651), or active season (β = 0.0763, *p* = 0.4876). Thus, none of the examined variables showed a statistically significant association with reported crop damage in this model.

### 3.9. Reported Economic Losses Associated with Asian Badger-Related Crop Damage

Among respondents reporting Asian badger-related crop damage, 16 provided monetary estimates of crop losses. The cumulative reported economic loss among these respondents was CNY 1710, corresponding to a mean reported loss of CNY 106.88 per respondent providing a monetary estimate. These monetary estimates represent only the subset of respondents who provided quantitative loss information and should not be interpreted as the total economic cost of Asian badger-related crop damage across the full survey sample. Respondents also suggested several potential non-lethal measures for reducing human–badger conflict, including regulated electric fencing, guard animals such as dogs, and visual or auditory deterrents.

### 3.10. Camera-Trap Detections, Relative Abundance Index, and Station-Level Detection Proportion of Asian Badgers

We conducted camera trapping at 82 stations across the four study areas from April 2024 to August 2026. The monitoring period comprised 275 sampling nights from April to December 2024, 365 nights from January to December 2025, and 243 nights from January to August 2026, totaling 883 sampling nights per camera and 72,406 trap-nights. We retained 103 independent photographic detections after applying the 30 min independence criterion. These detections confirmed Asian badger occurrence across multiple monitored locations within the Meng Mountains. The 103 independent detections were distributed among the four study areas as follows: Mingguang, 26 detections (21 cameras deployed); Mengyin, 36 detections (20 cameras deployed); Guimeng, 21 detections (21 cameras deployed); and Daqingshan, 20 detections (20 cameras deployed). Detection counts therefore varied descriptively among the study areas, with Mengyin contributing the largest number of independent records despite broadly similar camera allocation across sites. In addition, the questionnaire recall periods were not temporally identical to the full camera-trap monitoring period. A site-level summary of camera-trap deployment, operational trap-nights, independent Asian badger detections, questionnaire-reported encounters, and reported crop damage across the four study areas is presented in Table 14.

Reported encounters were based on Question 4 of the questionnaire; responses of daily, weekly, monthly, or rarely were classified as reported encounters, whereas “never” was classified as no reported encounter. Reported crop damage represents the damage attributed by respondents to Asian badgers and was not independently verified.

The camera-trap and questionnaire datasets covered the same four study areas and therefore permitted comparison at the study-site level. However, individual respondents or households were not spatially matched to individual camera-trap stations, and questionnaire responses were not paired temporally with specific camera-trap detections. Accordingly, the two datasets were analyzed separately and interpreted as complementary sources of ecological and human-dimension information rather than as a single spatially and temporally matched dataset.

The overall Relative Abundance Index (RAI) was 0.14 independent detections per 100 trap-nights. Detection frequency varied among camera-trap stations, with some stations recording multiple independent detections and others recording relatively few detections during the monitoring period. Asian badgers were detected at 52 of 82 targeted camera-trap stations, corresponding to a station-level detection proportion of 0.63. Because camera stations were deliberately placed in areas where badger occurrence was suspected, this proportion describes detections within the targeted monitoring network and should not be interpreted as landscape-wide occupancy.

### 3.11. Diel Activity Patterns of Asian Badgers

We evaluated diel activity patterns using 103 independent Asian badger detections from the camera-trap survey. We divided the 24 h cycle into diurnal, crepuscular, and nocturnal periods. Detection distributions differed significantly among these three periods (χ^2^ = 14.99, df = 2, *p* < 0.001). Detections occurred more frequently during diurnal and crepuscular periods, whereas comparatively fewer detections were recorded during nocturnal hours, indicating significant temporal variation in Asian badger detections across the daily cycle.

Selection-ratio analysis showed values greater than one during the diurnal ($w_{i}=1.17$) and crepuscular ($w_{i}=1.08$) periods, whereas the nocturnal period had a lower selection ratio ($w_{i}=0.76$). These values indicated greater use of diurnal and crepuscular periods relative to their temporal availability and lower use of nocturnal hours. Activity-pattern modeling also showed a bimodal temporal distribution, with one increase in the detections during daytime and another during twilight. Within the crepuscular period, detections were concentrated more strongly around sunset than around sunrise.

The estimated overall activity level was 0.446±0.053, corresponding to an estimated higher-activity period of approximately 10.7 h within a 24 h cycle. The remaining approximately 13.3 h represented periods of comparatively lower estimated activity. Overall, the camera-trap results showed that Asian badger detections were concentrated primarily during diurnal and crepuscular periods, with reduced detection frequency during nocturnal hours.

## 4. Discussion

This study considered information from a community survey alongside camera-trap monitoring to examine human–Asian badger (*Meles leucurus*) interactions in the agricultural landscapes of the Meng Mountains. The findings show that Asian badger occurrence and human–wildlife conflict are related but distinct components of the same socioecological system. Camera traps documented Asian badgers at a substantial proportion of monitored stations, whereas community surveys revealed marked spatial and temporal variation in reported encounters and conflict-related outcomes. Reported crop damage was associated with agricultural land ownership and livestock ownership, while familiarity, encounter-related variables, and reported activity periods explained little variation in crop-damage responses. At the same time, respondent familiarity was associated with encounter-related experience and perception that the Asian badger population had increased as a result of park protection. Together, these findings indicate that reported crop damage and conflict awareness in the Meng Mountains vary in relation to agricultural resources, livelihood characteristics, local experience, and human perceptions, rather than reflecting badger occurrence alone. This interpretation aligns with broader human–wildlife conflict research that emphasizes the interaction of ecological and social processes rather than wildlife abundance or occurrence alone [3,10,11,12].

### 4.1. Agricultural and Livestock Context of Reported Damage and Conflict Awareness

Agricultural land ownership was significantly associated with respondent-reported Asian badger-related crop damage, while livestock ownership was associated with both reported crop damage and conflict awareness. These findings indicate that agricultural and livestock-related livelihood characteristics were associated with respondents’ reported damage and awareness of conflict, but they do not demonstrate that individual households directly experienced conflict. Agricultural landscapes can provide wildlife with readily accessible food resources while simultaneously increasing the economic consequences of wildlife foraging. Similar patterns have been observed across other human–wildlife conflict systems, where the extent and configuration of agricultural land influence the probability that wildlife activity will result in livelihood losses [10,12,30].

The significant association between livestock ownership and conflict awareness should nevertheless be interpreted as an association and not evidence that livestock ownership caused badger conflict. The questionnaire did not establish livestock predation as the mechanism underlying this relationship. Livestock ownership may instead characterize households with greater agricultural involvement, more intensive use of rural landscapes, or greater exposure to areas also used by badgers. Consequently, agricultural and livestock characteristics should be interpreted as components of the broader human-use context associated with encounters, reported damage, and awareness of conflict.

The crop-damage regression provides an important complementary result. Familiarity, encounter time, active time, and active season did not significantly explain reported crop-damage responses, and the overall model accounted for only a small proportion of the observed variation. Thus, frequent encounters or familiarity with Asian badgers was not sufficient to explain whether respondents reported agricultural damage. This distinction parallels the conceptual separation between wildlife encounters and human–wildlife conflict: people may regularly observe wildlife without experiencing significant negative consequences, whereas conflict occurs when wildlife activities intersect with economically or socially important resources [3,31,32]. The results therefore support management approaches that identify when badger presence becomes economically consequential, rather than treating all areas of badger occurrence as equivalent conflict zones.

### 4.2. Spatial and Temporal Patterns of Human-Reported Encounters

Respondent-reported Asian badger encounters varied substantially among study sites, with Mingguang showing the highest reported encounter frequency and Mengyin the lowest. A notable contrast was observed in Mengyin, where reported encounter frequency was comparatively low despite a high proportion of respondents attributing crop damage to Asian badgers. Several non-exclusive factors could contribute to this discrepancy. Agricultural practices and crop composition may influence opportunities for crop damage independently of how often residents directly observe badgers, while the spatial distribution of respondents, cultivated fields, and badger-used habitats may also differ within the study area. In addition, questionnaire responses concerning damage and encounters may be affected differently by recall and perception. Because crop-damage attribution was respondent-based and was not independently verified, the available data cannot determine which of these mechanisms explains the Mengyin pattern. The discrepancy should therefore be interpreted as a site-specific pattern requiring further targeted investigation rather than as evidence that encounter frequency directly predicts crop damage. Significant differences also emerged among locations, reported sighting times, and sighting seasons. These spatial differences suggest that opportunities for human–badger encounters are not distributed uniformly across the Meng Mountains. Similar spatial heterogeneity has been documented in other human–wildlife conflict systems, including the Asiatic black bear study in Zhejiang, where conflict occurrence varied with topography, human disturbance, and proximity to wildlife habitat [12]. Such heterogeneity supports site-specific rather than landscape-wide uniform management, particularly where agricultural areas occur close to forested habitats.

Evening encounters were reported more frequently than encounters during other parts of the day, while autumn had the greatest number of seasonally reported encounters and winter the fewest. These questionnaire-derived patterns should be interpreted as human-reported encounter timing and seasonality, rather than as direct measurements of Asian badger activity. Human observations depend not only on animal behavior but also on when people are present outdoors, agricultural work schedules, visibility, recall, and the probability that an encounter is noticed. This distinction matters because camera-trap monitoring provides an independent measure of temporal detections and does not produce the same representation of daily activity as the questionnaire.

Seasonal concentration of reported encounters may nevertheless have practical relevance. Human–wildlife interactions commonly vary with seasonal changes in animal energetic requirements, food availability, agricultural phenology, and human land use. The human-bear study from Zhejiang similarly found strong seasonal clustering of conflict events and interpreted these patterns in relation to seasonal food availability and energetic demands [12]. In the present study, however, questionnaire data alone cannot identify the ecological mechanism behind greater autumn reporting. The most appropriate interpretation is therefore that autumn represented a period of greater reported human exposure to Asian badgers, which may help define periods when community awareness and crop-protection measures deserve greater attention.

### 4.3. Familiarity, Perceptions, and Conservation Awareness

Respondents differed substantially in their familiarity with Asian badgers, demonstrating that wildlife presence within a landscape does not necessarily produce uniform knowledge among local communities. Familiarity was positively associated with respondents’ perception that the Asian badger population had increased as a result of park protection and with encounter time. This association reflects respondents’ perceptions and should not be interpreted as evidence that park protection caused an actual increase in the Asian badger population. At the same time, the model examining public perceptions showed that familiarity was the only statistically significant predictor among the variables included. These relationships align with the broader human-dimension literature, which shows that direct experience with wildlife can shape species knowledge and perceptions [6,11,33,34]. People who encounter wildlife more frequently or interact regularly with landscapes used by wildlife may accumulate local ecological knowledge through observation and experience.

The positive relationship between familiarity and perceptions should not, however, be interpreted as evidence that familiarity automatically produces support for conservation or reduces conflict. The crop-damage model showed no significant association between familiarity and reported crop damage. Knowledge, attitudes, and economic impacts therefore appear to represent partially independent dimensions of human–badger relationships. The Human-Bear Conflict study provides a useful comparison: attitudes toward Asiatic black bears were strongly associated with livelihood exposure, with beekeepers who experienced direct economic losses showing particularly negative attitudes toward bears [12]. This shows why conservation awareness must be considered alongside the material costs households experience.

Community knowledge can nonetheless be valuable for conservation. Respondents provided information on encounter locations, timing, seasonality, perceived impacts, and possible coexistence measures that would be difficult to obtain solely through camera traps. These local observations complement ecological monitoring by showing where wildlife and human activities are perceived to overlap. However, questionnaire-based observations are influenced by memory and perception and should not be treated as interchangeable with independently collected ecological data. Several questionnaire items also relied on broad response categories without detailed operational definitions; consequently, responses may reflect variation in respondents’ interpretation of terms such as familiarity, local occurrence, and perceived impacts. These limitations mean that the questionnaire results should be interpreted as self-reported perceptions and experiences rather than as objective measurements of badger ecology or independently verified impacts. Because conflict awareness and crop damage were elicited through separate questionnaire items and were not cross-validated, the difference between the numbers reporting conflict awareness and crop damage may partly reflect differences in respondents’ interpretation of the broader concept of “conflict” and the more specific crop-damage item. Combining community observations with camera-trap monitoring, as undertaken here, therefore offers a stronger framework than either approach alone for understanding human–wildlife interactions [6,10,13,14].

### 4.4. Reported Crop Damage, Economic Losses, and Compensation

Reported crop damage differed among study sites and was associated with agricultural land and livestock ownership. Because attribution of crop damage to Asian badgers was based on respondents’ own interpretation and was not independently verified, some reported damage could potentially have been attributable to other species. Accordingly, these results should be interpreted as respondent-attributed crop damage rather than confirmed species-specific damage. These findings are consistent with the broader observation that the economic consequences of wildlife are often unevenly distributed within rural communities. Households whose agricultural resources overlap more strongly with wildlife-use areas may experience greater exposure to damage than households with little or no agricultural involvement. Comparable patterns have been reported for other conflict-prone mammals, where agricultural production and proximity to wildlife habitat influence the distribution of damage and economic costs [10,35,36,37].

Economic-loss information in the present study should be interpreted cautiously because only 16 respondents provided quantitative monetary estimates. Among this subset, cumulative reported losses amounted to CNY 1710, with a mean reported loss of CNY 106.88 per respondent providing a monetary estimate. These values show that measurable household-level costs were reported, but they do not estimate the total economic loss across all households reporting crop damage. Because the magnitude and form of wildlife-related losses are strongly species- and livelihood-specific, the monetary estimates reported here should be interpreted within the context of Asian badger-related crop damage in the Meng Mountains rather than directly compared with economic losses reported for biologically dissimilar wildlife species.

No significant association was detected between reported crop damage and receipt of compensation. This result does not show that compensation systems are ineffective; rather, the survey data provide no evidence that compensation status differed by reported crop-damage status. Compensation programs in human–wildlife conflict systems may face challenges related to eligibility requirements, damage verification, delayed payments, and administrative procedures [38,39,40,41]. Consequently, clearer damage documentation and accessible reporting mechanisms may be useful where compensation is intended to support households experiencing wildlife-related losses.

Respondents also suggested several non-lethal approaches to coexistence, including regulated electric fencing, guard animals, and visual or auditory deterrents. Similar physical and behavioral deterrents have been tested in other human–wildlife systems, although their effectiveness varies according to species, local conditions, maintenance, and habituation [2,42]. The bear paper provides a particularly relevant example: relocating beehives and using barriers were perceived as moderately effective, whereas repeated alarm-based deterrence appeared to lose effectiveness as bears habituated to the stimulus. Therefore, any non-lethal method recommended for Asian badgers should be evaluated locally rather than assumed effective because it worked with another species.

### 4.5. Asian Badger Occurrence and Diel Activity from Camera Traps

Camera trapping provided independent evidence that Asian badgers occurred across a substantial proportion of the surveyed landscape. Badgers were detected at 52 of 82 camera-trap stations, corresponding to a naïve occupancy of 0.63, while 103 independent detections were obtained during the monitoring period. The RAI was 0.14 independent detections per 100 trap-nights. Together, these metrics indicate relatively broad detection across monitoring locations while detection frequency at individual stations remained comparatively limited. This pattern is compatible with a mobile mesocarnivore that uses heterogeneous landscapes and may visit individual camera locations intermittently. Recent research on Asian badgers in northeastern China has likewise demonstrated their capacity to occupy heterogeneous habitats and emphasized the importance of landscape characteristics and suitable sett conditions in determining occurrence [8].

Naïve occupancy must be interpreted as observed site use rather than true occupancy because it does not account for imperfect detection. A site at which no badger was photographed cannot necessarily be assumed to have been unused by the species. This limitation is especially relevant because cameras in the present study were positioned in areas where preliminary surveys suggested badger presence. The resulting estimate therefore describes badger detection across the surveyed camera network rather than an unbiased estimate of landscape-wide occupancy. Nevertheless, the detections establish that Asian badgers persisted across multiple locations in a landscape containing forest habitat, agricultural areas, and human activities, supporting the value of integrating ecological monitoring with household-level information on wildlife interactions.

The diel activity results were particularly informative. Camera-trap detections differed significantly among diurnal, crepuscular, and nocturnal periods, with selection ratios greater than one for diurnal (w_i_ = 1.17) and crepuscular (w_i_ = 1.08) periods and below one for nocturnal periods (w_i_ = 0.76). The estimated activity curve was bimodal, with peaks during daytime and twilight, and crepuscular detections were concentrated more strongly around sunset than sunrise. Rather than demonstrating a general shift in Asian badger activity patterns, these data document a greater representation of diurnal and crepuscular detections within the Meng Mountains during the study period.

Diel detection patterns in mesocarnivores can vary with human disturbance, food availability, habitat characteristics, temperature, and other ecological conditions. Camera-trap research in the Taihang Mountains of central North China showed that Asian badgers reduced daytime activity under greater human disturbance, suggesting that activity timing can respond to anthropogenic pressure [9]. Our greater representation of diurnal and crepuscular detections may therefore reflect local conditions within the Meng Mountains, although the present data do not identify the mechanism responsible. Accordingly, the observed pattern should not be generalized as a species-wide change in Asian badger activity.

### 4.6. Conservation and Management Implications

Effective human–wildlife conflict mitigation requires management actions targeted to the locations, periods, and livelihood contexts where negative interactions are most likely to occur [10,12,43]. In the present study, integrating community-reported encounters and damage with camera-trap information on Asian badger occurrence and activity provides a basis for such targeted management. The results indicate that management should focus primarily on places and households where Asian badger occurrence overlaps with agricultural resources rather than on wildlife presence alone.

**Figure 10:**
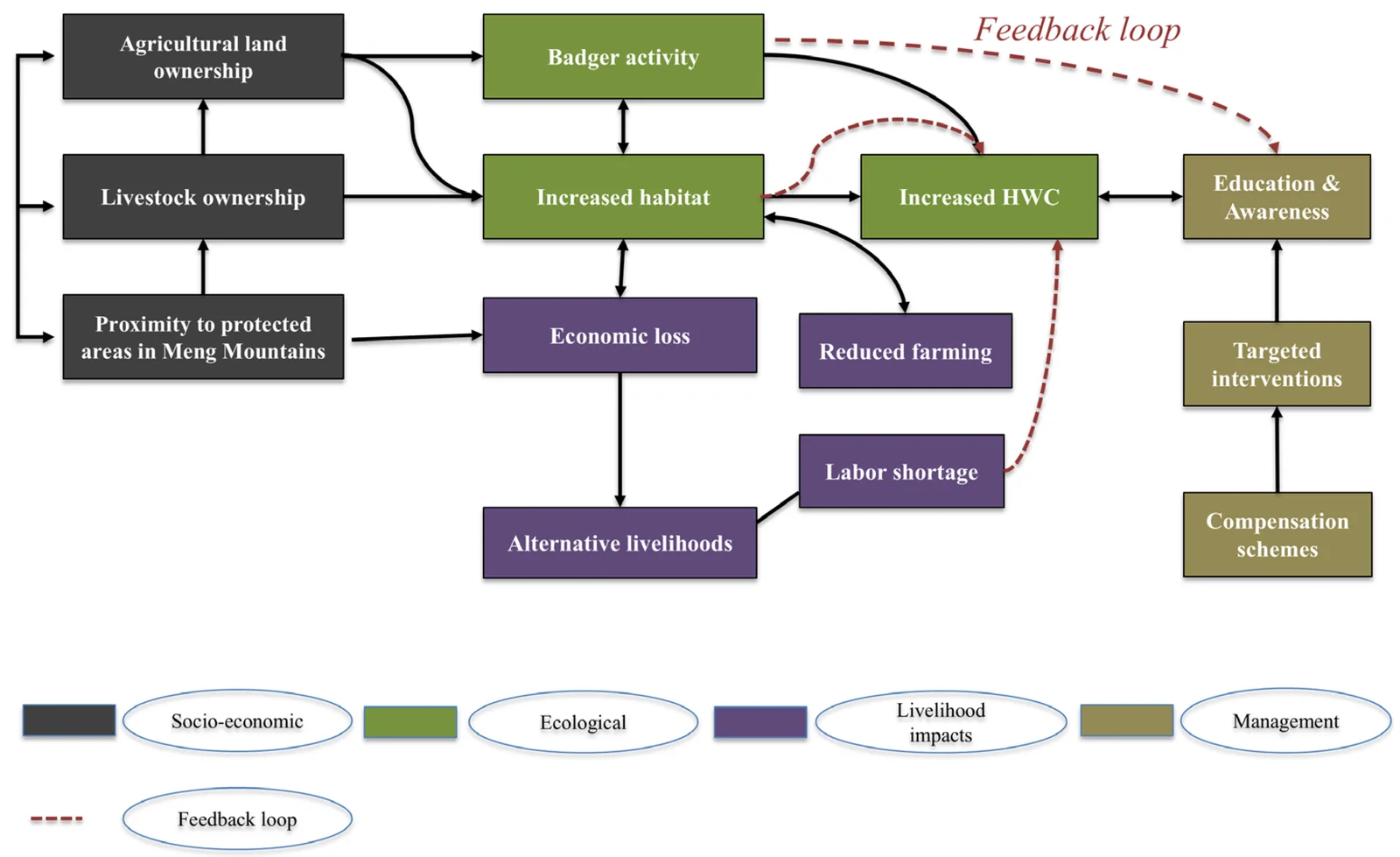
Conceptual framework of human–Asian badger interactions and conflict in agricultural landscapes. The framework illustrates potential linkages among socioeconomic factors (dark gray), ecological factors (green), household and livelihood consequences (purple), and management responses (brown). Socioeconomic components include agricultural land ownership, livestock ownership, and relationships with protected areas, whereas ecological components include habitat conditions and Asian badger activity. Household-level consequences include economic losses and potential livelihood responses, while management components include compensation, targeted interventions, and awareness-raising. Solid black arrows indicate the proposed directional relationships among socioeconomic, ecological, livelihood, and management factors within the conceptual framework. Dashed arrows indicate potential feedback pathways among land use, habitat conditions, and human–wildlife interactions. The framework integrates findings from the present study with broader conceptual pathways relevant to human–wildlife conflict; not all pathways shown were directly tested statistically in this study.

Based on the combined ecological and social findings, we propose a conceptual framework illustrating the relationships among ecological conditions, socioeconomic characteristics, household-level consequences, and management responses relevant to human–Asian badger interactions in the Meng Mountains (Figure 10). The framework places the observed associations identified in this study within a broader human–wildlife conflict context. It illustrates how wildlife activity, agricultural exposure, livelihood consequences, and management responses may interact. Some pathways shown in the framework represent broader conceptual relationships rather than effects directly tested in the present study.

Several practical implications follow. Agricultural households and locations with recurrent reported damage may warrant greater attention for crop-protection measures, particularly during periods of greater reported encounters. Measures suggested by respondents, such as regulated electric fencing, guard animals, and visual or auditory deterrents, could be considered within a non-lethal mitigation framework, provided their effectiveness and animal-welfare implications are evaluated locally. Preventive approaches are preferable to indiscriminate population reduction because Asian badgers also contribute ecological functions within terrestrial communities and agricultural mosaics [44,45].

Temporal information can further improve targeting. Questionnaire responses identified evening and autumn as periods of increased reported encounters, while camera traps demonstrated substantial diurnal and crepuscular activity, including elevated detections around sunset. Consequently, mitigation efforts should not assume that Asian badgers are exclusively nocturnal. Where crop protection or monitoring is feasible, greater attention around twilight and other locally identified encounter periods may reduce opportunities for negative interactions. This approach follows the precautionary management logic of the wild-boar study, which used knowledge of wildlife distribution and activity to determine when and where to concentrate interventions [10].

Community engagement should accompany physical mitigation. Familiarity was associated with respondents’ perceptions of Asian badgers, suggesting that local experience and knowledge contribute to the social context of coexistence. Education programs can clarify badger ecology, ecological functions, and appropriate responses to encounters, but education alone should not be expected to resolve conflict where households continue to experience material losses. The most appropriate strategy is therefore integrated, combining ecological monitoring, targeted damage prevention, community participation, improved documentation of losses, and transparent compensation or support mechanisms where appropriate [3,6,11,31,32,38].

## 5. Conclusions

This study combined community surveys and camera-trap monitoring as complementary approaches to examine the ecological and human dimensions of Asian badger (*Meles leucurus*) interactions in the agricultural landscapes of the Meng Mountains, China. Human-reported encounters, crop damage, economic impacts, familiarity, and perceptions varied among respondents and study sites, demonstrating that human–badger interactions were spatially and socially heterogeneous. Agricultural land ownership was significantly associated with reported crop damage, while livestock ownership was associated with both reported crop damage and conflict awareness. In contrast, familiarity and the examined encounter-related temporal variables explained little variation in reported crop-damage responses, indicating that wildlife exposure alone did not account for reported agricultural impacts. Camera-trap monitoring provided an independent ecological perspective on Asian badger occurrence and activity. Asian badgers were detected at 52 of 82 camera-trap stations, corresponding to a naïve occupancy of 0.63, and we recorded 103 independent detections during the monitoring period. Detections were concentrated more strongly during diurnal and crepuscular periods than during nocturnal hours, and the estimated activity pattern was bimodal. These findings complement the questionnaire results by distinguishing independently recorded badger activity from human-reported encounter patterns. Together, the two data sources show that Asian badgers occur across a substantial proportion of the monitored landscape. In contrast, negative human–badger interactions are more closely associated with circumstances where wildlife use overlaps with agricultural resources and livelihood activities.

The findings support a targeted coexistence approach rather than uniform management across the landscape. Conflict mitigation should prioritize households and locations experiencing recurrent agricultural impacts and combine non-lethal crop-protection measures, community engagement, improved documentation of wildlife-related losses, appropriate compensation mechanisms, and continued ecological monitoring. Integrating camera-trap information on occurrence and temporal activity with community-based information on encounters, perceptions, and livelihood impacts provides a practical framework for reducing human–Asian badger conflict while maintaining the ecological role of the species in the Meng Mountains.

## Figures and Tables

**Table 1:** Demographic overview of survey respondents (N = 400).

Variable	Description
Total respondents	400
Study sites	Mingguang, Mengyin, Guimeng, Daqing Shan
Respondent sex	Men, 54%; women, 46%
Agricultural land ownership	82.75%
Livestock ownership	12.0%
Mean agricultural land area	2.5 ha

**Table 2:** Ordinal logistic regression of factors associated with respondents ’ self-reported familiarity with Asian badgers (N = 400).

Predictor	Coefficient	Std. Error	*p*-Value
Perceived population increase associated with park protection	1.788	0.270	<0.001
Encounter time	2.155	0.192	<0.001
Active time	0.275	0.146	0.059

**Table 3:** Spearman correlations between conflict awareness conflict and familiarity, encounter time, and season.

Variable	Spearman’s ρ	*p*-Value	N
Familiarity	−0.002	0.964	400
Encounter time	−0.021	0.676	400
Active season	−0.068	0.178	394

**Table 4:** Associations between agricultural land ownership, livestock ownership, and reported Asian badger-related crop damage among respondents (N = 400).

Predictor Variable	Category	Total	χ^2^	df	*p*-Value	Cramér’s V
Agricultural land ownership	Forest land	5				
	No land	64				
	Agricultural land	331	16.6436	2	0.0002	0.2040
Livestock ownership	0 animals	352				
	1 animal	30				
	2 animals	7				
	3 animals	1				
	4 animals	3				
	6 animals	3				
	10 animals	2				
	15 animals	1				
	20 animals	1	36.2265	8	<0.001	0.3009
Total (N)		400				

**Table 5:** Distribution of valid respondent-reported Asian badger encounter observations among seasons (N = 394).

Season	Number of Valid Responses (N)
Spring	40
Summer	144
Autumn	209
Winter	1
Total valid seasonal responses	394

**Table 6:** Chi-square test assessing spatial variation in respondent-reported Asian badger encounters among sighting locations (N = 400).

Statistic	Value
Chi-square (χ^2^)	139.3573
Degrees of freedom (df)	6
*p*-value	<0.001
Sample size (N)	400
Cramér’s V	0.4174

**Table 7:** Multiple linear regression examining respondent-reported Asian badger encounter frequency in relation to encounter time, active time, and active season (N = 394).

Predictor Variable	Coefficient	t-Value	*p*-Value
Constant	0.5050	9.7871	<0.001
Active time	0.0153	0.6894	0.4910
Active season	−0.0575	−2.0357	0.0425
Encounter time	0.2729	12.4645	<0.001

**Table 8:** Chi-square tests assessing variation in respondent-reported Asian badger encounters across spatial and temporal categories.

Habitat Variable	Chi-Square (χ^2^)	*p*-Value
Location	139.357	<0.001
Time of sighting	16.007	0.0137
Season of sighting	13.854	0.0313

**Table 9:** Ordinal logistic regression examining factors associated with respondents’ self-reported familiarity with Asian badgers.

Predictor Variable	Coefficient	Std. Error	z-Value	*p*-Value	95% CI Lower	95% CI Upper
Perceived population increase associated with park protection	1.7884	0.2703	6.6170	<0.001	1.2586	2.3181
Encounter time	2.1547	0.1924	11.1980	<0.001	1.7776	2.5318
Active time of day	0.2752	0.1456	1.8905	0.0587	−0.0101	0.5605
Threshold (0/1)	3.2662	0.4665	7.0016	<0.001	2.3519	4.1805
Threshold (1/2)	1.3423	0.0744	18.0388	<0.001	1.1964	1.4881

**Table 10:** Ordinal logistic regression examining factors associated with respondents’ perceptions of Asian badgers.

Predictor Variable	Coefficient	Std. Error	z-Value	*p*-Value	95% CI Lower	95% CI Upper
Familiarity	1.8376	0.2793	6.5786	<0.001	1.2901	2.3851
Encounter time	0.3289	0.2308	1.4247	0.1542	−0.1235	0.7813
Active time of day	0.2240	0.1836	1.2203	0.2224	−0.1358	0.5837
Threshold (0/1)	−1.7255	0.3893	−4.4324	<0.001	−2.4884	−0.9625
Threshold (1/2)	1.1330	0.1075	10.5428	<0.001	0.9224	1.3436

**Table 11:** Chi-square test of association between livestock ownership and conflict awareness (N = 400).

Statistic	Value
Chi-square (χ^2^)	60.474
Degrees of freedom (df)	8
*p*-value	<0.001
Sample size (N)	400
Cramér’s V	0.389

**Table 12:** Observed frequencies of conflict awarenessacross livestock-ownership categories.

Livestock Owned	Not Aware of Conflict	Aware of Conflict	Total
0	260	92	352
1	4	26	30
2	1	6	7
≥3	10	1	11
Total	275	125	400

Note: The chi-square test reported in Table 11 was performed using the nine original livestock-count categories (0, 1, 2, 3, 4, 6, 10, 15, and 20 animals). For descriptive presentation in Table 12, categories of three or more animals were combined as ≥3.

**Table 13:** Multiple linear regression examining factors associated with reported Asian badger-related crop damage (N = 195). The reported N = 195 represents the effective sample available for the response variable and all predictors included in this model.

Variable	Coefficient	Std. Error	t-Value	*p*-Value	95% CI Lower	95% CI Upper
Constant	1.3758	0.2063	6.6673	<0.001	0.9687	1.7828
Familiarity	−0.1382	0.1339	−1.0326	0.3031	−0.4022	0.1258
Encounter time	−0.0804	0.1125	−0.7147	0.4756	−0.3024	0.1415
Active time	0.0230	0.0770	0.2992	0.7651	−0.1288	0.1749
Active season	0.0763	0.1098	0.6954	0.4876	−0.1402	0.2928

Model summary: R^2^ = 0.0215, adjusted R^2^ = 0.0009, *p* = 0.386, N = 195.

**Table 14:** Site-level summary of camera-trap monitoring and questionnaire-reported Asian badger interactions across the four study areas.

Study Site	Cameras Deployed	Operational Trap-Nights	Independent Badger Detections	Reported Encounters, n (%)	Reported Crop Damage, n (%)
Mingguang	21	18,543	26	62 (62%)	26 (26%)
Mengyin	20	17,660	36	25 (25%)	94 (94%)
Guimeng	21	18,543	21	55 (55%)	31 (31%)
Daqingshan	20	17,660	20	97 (97%)	48 (48%)
Total	82	72,406	103	239 (59.75%)	199 (49.75%)

## Data Availability

Data generated and/or analyzed during this study are available from the corresponding author on reasonable request.

## Supplementary Materials

The following supporting information can be downloaded at: https://www.mdpi.com/article/10.3390/ani16203294/s1, S1: Questionnaire.
