# Supplementary material for: Integrating Camera-Trap Monitoring and Community Surveys to Assess Human–Asian Badger (Meles leucurus) Interactions in Agricultural Landscapes of the Meng Mountains, China
Source: Animals (Basel). 2026 Oct 19;16(20):3294. doi: 10.3390/ani16203294 (PMC13700675; doi:10.3390/ani16203294)
Supplement: Supplementary file 1 [file animals-16-03294-s001.zip › animals-4597076-supplementary.pdf]

Questionnaire

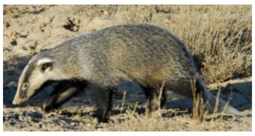

Integrating Camera-Trap Monitoring and Community Surveys to Assess Human-Asian Badger (*Meles leucurus*) Interactions in Agricultural Landscapes of the Meng Mountains, China.

Date ______________________________
Name & Age ________________________
Village Name _______________________
Education _________________________

Ethnic background ___________________
Occupation ________________________
Agricultural land ____________________

1. How familiar are you with the Asian badger (*Meles leucurus*)?

Very familiar	Somewhat familiar	Not familiar at all

2. Have you ever seen an Asian badger in the wild? Yes No

3. Where have you seen badgers in the Meng Mountains? Number of individuals in a single encounter (Please provide specific locations)

____

4. How frequently do you encounter badgers in your area?

Daily Weekly Monthly Rarely Never

5. What time of day do you most often observe badgers? (Select all that apply)

Morning Afternoon Evening Night

6. During which season do you notice more badger activity?

Spring Summer Autumn Winter

7. Are you aware of any conflicts between badgers and humans in your area? (e.g., property damage, livestock predation)

Yes No

8. Did you see any of the following species (e.g., feeding, observing)?

Species	Asian badger	Masked Palm Civet	Hare	Other wild animals
Numbers				
Status (Common/Rare/Absent)				

9. Did the badger population increase as a result of Protection within the park?

Species	Yes	No	Don't know
Asian badger			

10. How many livestock animals does your household own? Have your livestock been vaccinated? (Yes/No)

Livestock	Goats	Sheep	Cattle	Equine/others
Number				
Vaccinated				

11. Crops damaged by Asian badger in the past 1 year:

Season	Winter	Spring	Summer	Autumn
Crop type (peanut, corn, bean, apple, peach)				
Loss (RMB)				

12. Have you received any compensation from Govt. & Non-Govt organizations? If yes, how much of the total loss?

13. Do you pay for grazing of livestock within the boundary of the National Park?

14. Do you think there is any possible solution for human-badger coexistence?

15. Please provide any additional comments or observations regarding badgers in your area.
